# Differentiable optimization layers enhance GNN-based mitosis detection

**DOI:** 10.1038/s41598-023-41562-y

**Published:** 2023-08-31

**Authors:** Haishan Zhang, Dai Hai Nguyen, Koji Tsuda

**Affiliations:** 1 Department of Computational Biology and Medical Sciences, Graduate School of Frontier Sciences, The University of Tokyo, Kashiwa, Chiba 277-8561 Japan; 2 Department of Computer Science, The University of Tsukuba, 1-1-1 Tennodai, Tsukuba, Ibaraki 305-8577 Japan; 3 RIKEN Center for Advanced Intelligence Project, 1-4-1 Nihonbashi, Chuo-ku, Tokyo 103-0027 Japan; 4 Research and Services Division of Materials Data and Integrated System, National Institute for Materials Science, Tsukuba, Ibaraki 305-0047 Japan

**Keywords:** Image processing, Mitosis

## Abstract

Automatic mitosis detection from video is an essential step in analyzing proliferative behaviour of cells. In existing studies, a conventional object detector such as Unet is combined with a link prediction algorithm to find correspondences between parent and daughter cells. However, they do not take into account the biological constraint that a cell in a frame can correspond to up to two cells in the next frame. Our model called GNN-DOL enables mitosis detection by complementing a graph neural network (GNN) with a differentiable optimization layer (DOL) that implements the constraint. In time-lapse microscopy sequences cultured under four different conditions, we observed that the layer substantially improved detection performance in comparison with GNN-based link prediction. Our results illustrate the importance of incorporating biological knowledge explicitly into deep learning models.

## Introduction

**Figure 1:**
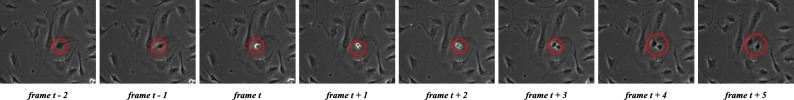
Example of cell mitosis. A parent cell divides into two daughter cells at frame *t*.

With recent advances in imaging techniques, tracking cell nuclei in time-lapse living cell microscopy images has become possible in the studies of developmental biology^1^. The cell tracking problem^2,3^ is different from general object tracking^4,5^ due to cell mitosis, i.e., a cell divides into two daughter cells (Fig. 1). Existing computer vision studies about mitosis detection can be classified into two categories: shape-based and link-based. In shape-based detection^6,7^, each frame of the video is treated separately and cells with the characteristic shape, i.e., spherical and bright bordered, are detected as parent cells by conventional deep learning detectors. Note that the characteristic shape is specific to certain types of medium and microscopies. In link-based detection^8,9^, cells of all shapes are detected and the links between the cells in neighboring frames are predicted. Subsequently, cells with two outgoing links are identified as parents. An advantage of link-based methods over shape-based ones is that daughter cells can be identified as well. Jug et al.^8^ proposed to detect cells by random forest and construct links by mathematical programming. An advantage of mathematical programming is that biological constraints, i.e., a cell is divided to at most two cells, can be explicitly taken into account. More recently, Ben-Haim and Raviv^9^ proposed a graph neural network (GNN)^10^-based method for inferring cell links including mitosis. While it outperformed other methods such as AGC^11^, ST-GDA^12^, BFP^13^ and MPM^14^, the constraints are not imposed. In this paper, we develop a deep neural network that explicitly takes the constraint into account via differentiable optimization^15^.

A deep neural network (DNN) is a function with multidimensional input and output vectors. It is a composite function of multiple functions called *layers*, where the output vectors of upstream layers are fed to downstream layers. Layers have a number of parameters that are optimized to minimize a given loss function dependent on the training examples. Parameter optimization is done by computing the derivative of the loss function with respect to the parameters with back propagation. Namely, the derivatives of downstream layers are computed first and those of upstream layers are computed using downstream derivatives based on the chain rule. Layers are implemented by diverse kinds of computation including convolution^16^, attention^17^ and message passing^18^. DNNs have been applied to various domains^19–22^. Amos and Zico^15^ proposed to employ a mathematical program as a layer, that is, the coefficients and solution of the program are designated as input and output, respectively. To enable back propagation, the derivative of the solution with respect to the coefficients is computed based on Karush–Kuhn–Tucker (KKT) condition and implicit function theorem. This layer is called *differentiable optimization layer* (DOL). An advantage of DOL is that a hard constraint can be imposed on the output. In the following, we use DOL to impose the biological constraint of mitosis.

**Figure 2:**
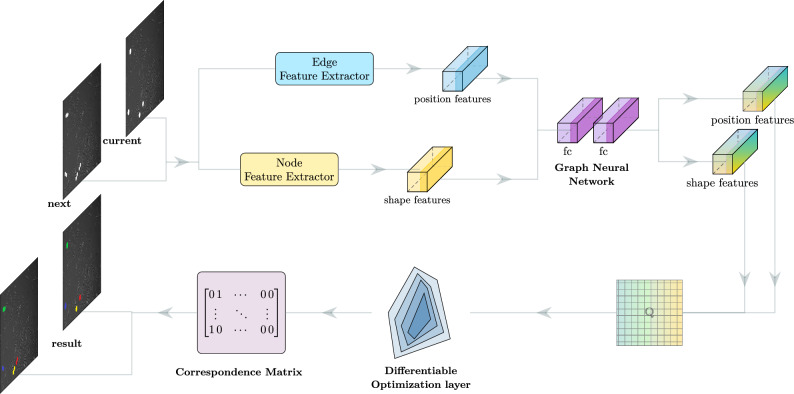
Overview of GNN-DOL. Cells are detected from each image and converted to a cell graph. Edge and node features are updated by a graph neural network (GNN), and the similarity matrix *Q* is constructed from the updated features. A differential optimization layer (DOL) derives the optimal correpondence between the cells from *Q*.

Our method assumes that all cell positions at frame *t* and $t+1$ are readily identified, e.g., by Unet^16^. Next, a cell graph is made for each frame by designating the identified cells as nodes and connecting neighboring nodes by edges. A node feature vector contains visual features and an edge feature vector has positional features. Our DNN has two blocks as shown in Fig. 2. The upstream one, a GNN, converts the two cell graphs into a similarity matrix *Q* and the downstream one, a DOL, takes *Q* as the coefficient of a quadratic program and produce the cell correspondence matrix across the frames. If a cell at frame *t* corresponds to two cells in frame $t+1$, it is identified as a parent cell.

Our method is evaluated on public C2C12 dataset, which contains time lapse microscopy image sequences. In addition to the original annotations of cell identities, extra annotations by Su et al.^7^ were used. To measure the impact of DOL, we implemented a naive GNN that predicts the correspondence matrix by a multilayer perceptron (MLP) layer that does not impose the constraint. In experiments, we observed that our method enhances the accuracy of mitosis detection in comparison to the naive GNN and GNN by Ben-Haim and Raviv^9^, demonstating that the use of DOL affected the upstream parameters of GNN in a favorable manner. A general tendency of deep learning approaches is to avoid hard constraints that describe the rules and let the model learn the rules from a large amount of data^23^. In comparison to general image data, however, scientific image data are smaller in amount by orders of magnitude^1^. Our results show that incorporating biological constraints via DOL is effective in scientific domains and our approach may be applicable to other scientific problems.

## Method

### Preprocessing by U-net

As training data, the positions of all cells at all frames and the correspondence between cells at neighboring frames are given. Each cell corresponding to two cells in the next frame is labeled as a parent cell. First, we build a predictor of cell positions using a deep neural network called U-net^16^. The input of U-net is an image and the output is a segmentation map, i.e., the image of the same size where all cell centroids are marked as bright pixels. U-net has a network architecture that first contracts an input image to a latent vector gradually with multiple layers and then expanding it to the output image. By applying U-net to a test image after training, we can predict the position of all cells. In addition, the shape feature vector of the image patch including each cell can be extracted from one of the contraction layers.

### GNN-DOL

Using the information from trained U-net, we construct a parent cell predictor that judges if a cell is a parent or not based on *cell graphs*. The *i*-th cell at frame *t* is denoted as $(x_i,y_i,{\textbf{v}}_i), i \in [1,m]$, where $(x_i,y_i)$ denote the position and ${\textbf{v}}_i$ is the shape feature vector. Similarly, those at frame $t+1$ are denoted as $(x_i^\prime , y^\prime _i, {\textbf{v}}^\prime _i), i \in [1,m^\prime ]$. The cell graph for frame *t* is constructed by designating the *m* cells as nodes and connecting each cell to two nearest neighbors among the cells by edges. Each node is labeled by ${\textbf{v}}_i$ and each edge is labeled by positional feature vector, ${\textbf{e}}_{ij} = (x_s,y_s,x_t,y_t)$, where $s,t (s<t)$ indicate the end nodes.

Our method GNN-DOL takes two cell graphs as input and provides the cell correpondence matrix as output. First, node features and edge features are updated via message passing^18^. Let *N*(*i*) denote the adjacent nodes of *i*. The node feature vector is updated using a multilayer perceptron (MLP) as

$$\begin{aligned} {{\bar{{\textbf{v}}}}}_i = \textrm{MLP}({\textbf{v}}_i, \sum _{j=1}^m w_{ij} {\textbf{e}}_{ij}) \end{aligned}$$

where

$$\begin{aligned} w_{ij} = \sum _{k \in N(i)} \sum _{l \in N(j)} \Vert {\textbf{e}}_{ik} - {\textbf{e}}_{jl} \Vert ^2. \end{aligned}$$

The edge feature vector is updated as

$$\begin{aligned} {{\bar{{\textbf{e}}}}}_{ij} = \textrm{MLP}({\textbf{e}}_{ij}, {\textbf{v}}_i, {\textbf{v}}_j). \end{aligned}$$

A MLP with two fully connected layers is used for both updates. Let ${{\bar{{\textbf{v}}}}}_k^\prime$ and ${{\bar{{\textbf{e}}}}}_{kl}^\prime$ denote the updated feature vectors from frame $t+1$. An $m m^\prime \times m m^\prime$ pairwise similarity matrix *Q* is derived as follows. Let $\alpha (i,j) = im+j$ and $\beta (k,l) = k m^\prime +l$. Also let *E* and $E^\prime$ denote the set of edges in the cell graphs. Then, *Q* is described as

$$\begin{aligned} Q_{\alpha \beta } = \left\{ \begin{array}{ll} {{\bar{{\textbf{v}}}}}_i ({{\bar{{\textbf{v}}}}}_k^\prime )^\top &{}\quad i=j, k=l \\ {{\bar{{\textbf{e}}}}}_{ij} ({{\bar{{\textbf{e}}}}}^\prime _{kl})^\top &{}\quad (i,j) \in E, (k,l) \in E^\prime \\ 0 &{}\quad otherwise. \end{array} \right. \end{aligned}$$

Let *Z* denote a $m \times m^\prime$ correspondence matrix, and ${\textbf{z}}= \textrm{vec}(Z)$. The quadratic program implemented in our DOL is described as

1 $$\begin{aligned} {\textbf{z}}^* = \textrm{argmin} \frac{1}{2} {\textbf{z}}^\top Q {\textbf{z}}, \; \mathrm{s.t.} \; A_{1} {\textbf{z}}= 1, \; A_2 {\textbf{z}}\le 2, \; 0 \le {\textbf{z}}\le 1 \end{aligned}$$

where $A_1 \in {\mathbb {R}}^{m\times m m^\prime }$ and $A_2 \in {\mathbb {R}}^{n \times m m^\prime }$ are defined as

2 $$\begin{aligned} A_1 = \begin{bmatrix} 1&{}\cdots &{}1&{} &{} &{} &{} &{} &{} \\ &{} &{} &{}1&{}\cdots &{}1&{} &{} &{} \\ &{} &{} &{} &{} &{} &{}1&{}\cdots &{}1 \end{bmatrix}, \; A_2 = \begin{bmatrix} I&I&\cdots&I \end{bmatrix}. \end{aligned}$$

The first constraint in (1) ensures that a cell at frame $t+1$ correponds to a cell at frame *t*. The second one is related to mitosis, i.e., a cell at frame *t* can correpond to at most two cells at frame $t+1$. From the optimial solution ${\textbf{z}}^*$, the correspondence matrix is obtained by taking column-wise maximum. If the cell of interest in frame *t* is connected to two cells in frame $t+1$, it is predicted as a parent. Training of GNN-DOL is implemented by Pytorch and neural-scs python package^24^ on a NVIDIA Tesla V100 GPU (32GB). We adopted the weighted binary cross entropy loss and used Adam optimizer with learning rate $1.0 \times 10^{-4}$. In applying the trained network to an image sequence, CVXPY package^25^ was used to solve the quadratic program. The detailed algorithm of GNN-DOL is shown in Supplementary Information.

### Naive GNN

For measuring the effect of DOL, we prepared another model called *naive GNN*, where the updated features are fed into a MLP whose output is the correspondence matrix ${\textbf{z}}$. Note that no constraints are imposed to ${\textbf{z}}$ here and the absence of DOL affects upstream GNN parameters via back propagation.

## Results and discussion

**Figure 3:**
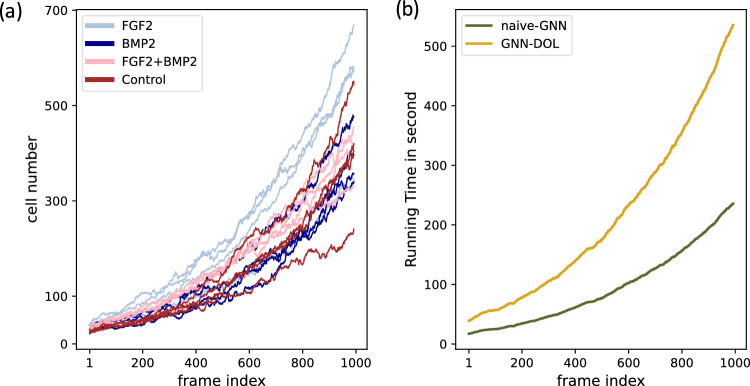
(**a**) Number of cells in each frame. (**b**) Computational time of GNN-DOL and Naive-GNN for processing a pair of consective frames.

We carry out all experiments on the public dataset, C2C12^26^, which contains time-lapse microscopy image sequences cultured under 4 different media conditions, including with fibroblast growth factor 2 (FGF2), bone morphogenetic protein 2 (BMP2), FGF2 + BMP2, and control (no growth factor). Each image sequence is composed of 1013 frames with size of 1392 $\times$ 1040 pixels. The images were recorded by Zeiss Axiovert T135V microscope with the resolution of 1.3 $\upmu$m/pixel. The cell displacement between two adjacent frames is about 6 pixel on average. The annotation of C2C12 consists of the coordinates of cell centroids and their corresponding cell identifiers. In the original distribution of C2C12, only one sequence, F0009, was annotated. We also used additional annotation covering all sequences contributed by Su et al.^7^. For each media condition, three sequences are used as training examples and the remaining one sequence is used as test examples (Table 1). Fig. 3a shows the number of cells at each frame. Cells may enter or exit the field of view, but the number of cells was consistently increasing due to mitosis.

For preprocessing for GNN-DOL, UNet is trained with the sequence F0009, and shape feature vectors are extracted from the contraction layer corresponding to 50 $\times$ 50 image patches. To accelerate the training of GNN-DOL, the number of cells is reduced to 30 as follows. For frame $t+1$, all the daughter cells are included first. The rest is filled with randomly chosen cells. For frame *t*, we include the parent cells of the daughter cells and the predecessors of the randomly chosen cells. Finally, additional cells are chosen at frame *t* to adjust the number of cells to 30. Notice that all cells are included in cell graphs in applying the trained GNN-DOL to test examples.

**Table 1:** C2C12 image sequences and their split into training and test examples.

Condition	Type	Sequences	Number of mitosis events
Group 1 (FGF2)	Training	F0001, F0002, F0003	1951
	Test	F0004	289
Group 2 (BMP2)	Training	F0005, F0006, F0007	1488
	Test	F0008	469
Group 3 (FGF2 + BMP2)	Training	F0009, F0010, F0011	1110
	Test	F0012	249
Group 4 (Control)	Training	F0013, F0014, F0015	1333
	Test	F0016	270

### Mitosis detection

We compared GNN-DOL with a state-of-the-art GNN model by Ben-Haim and Raviv (GNN-B & R)^9^. This model was trained only with F0009 in the original paper, but we trained it with all the annotations using their code^27^. Note that GNN-B & R does not impose the constraints on cell correspondences. GNN-DOL was additionally compared with MPM^14^, a popular mitosis detection method. We evaluated how accurately mitosis events (i.e., the parent cells) are identified in each test sequence (Table 2). It is observed that GNN-DOL performs consistently better than GNN-B & R, MPM and Naive-GNN. GNN-B & R achieved high precision but relatively low recall, indicating that they are likely to miss hard-to-detect mitosis events. Our results suggest that imposing the constraint by DOL is beneficial in identifying mitosis events correctly. A downside of using DOL, however, is that it is computationally more demanding due to the use of quadratic programming. Figure 3b shows that the computational time of GNN-DOL for processing a pair of frames is about three times longer than Naive-GNN.

**Table 2:** Detection accuracy of mitosis events.

Metric	Method	Group name			
		FGF2	BMP2	FGF2+BMP2	Control
F1-score	GNN-DOL	**0.869**	**0.815**	**0.792**	**0.811**
	GNN-B & R	0.653	0.646	0.615	0.688
	MPM	0.647	0.618	0.596	0.730
	Naive-GNN	0.515	0.591	0.589	0.473
Precision	GNN-DOL	0.940	0.945	0.950	0.957
	GNN-B & R	0.919	0.956	0.870	0.947
	MPM	0.716	0.508	0.601	0.823
	Naive-GNN	0.551	0.676	0.686	0.698
Recall	GNN-DOL	0.808	0.717	0.679	0.704
	GNN-B & R	0.506	0.487	0.476	0.540
	MPM	0.590	0.790	0.592	0.656
	Naive-GNN	0.483	0.525	0.516	0.357

The highest F1-scores are in bold.

### Cell correspondence prediction

**Figure 4:**
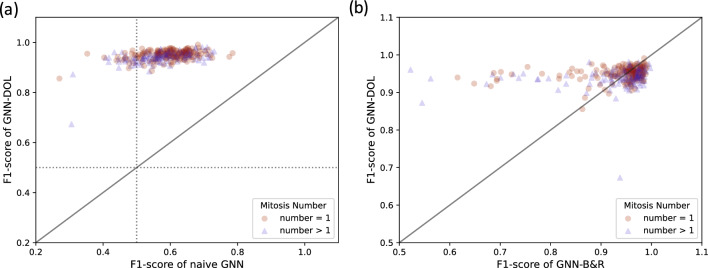
(**a**) Distribution of F1-scores of GNN-DOL and Naive-GNN for identifying cell correspondences. The horizontal and vertical axes represent the F1-scores of Naive-GNN and GNN-DOL, respectively. Marker shapes indicate the number of mitosis events in a frame. A circular marker shows that the frame has only one event and a triangular one shows that there are two or more. (**b**) Comparison of GNN-DOL and GNN-B & R.

We also investigated the prediction accuracy of cell correpondence prediction. Frames with at least one parent cell and their next frames are chosen for examination. GNN-DOL, GNN-B & R and Naive-GNN are applied to the pair of cell graphs derived from the frame pairs. The predicted corresponding matrix *Z* is compared with the ground truth. In Fig. 4a,b GNN-DOL is compared with GNN-DOL and GNN-B & R in terms of F1-score, respectively. GNN-DOL performed better than Naive-GNN and GNN-B & R consistently, showing the significant contribution of DOL. It is also found that GNN-B & R tends to fail badly, when there are more than one mitosis events happening. Multiple mitosis cases occur rarely in training data, so it should be hard to learn via purely data-driven approaches.

## Conclusion

In this paper, we proposed a differentiable model, GNN-DOL, to detect cell mitosis from video. GNN-DOL was particularly accurate, when there are multiple mitosis events in an image. Our method should be useful to measure mitosis frequency under the influence of a drug, which is an important step of drug development. While GNN-DOL is designed for predicting cell correspondences in two adjacent images and amenable to online video processing, it may also contribute to cell lineage analysis^28,29^ over a long time frame. A drawback of our method is that U-net and GNN-DOL are separate and not unified. The cell identification mistakes of U-net are carried over to GNN-DOL and can never be corrected. If a unified network that can learn the mitosis detection task in the end-to-end fashion is constructed, this problem may be alleviated.

Deep learning models have shown that, given a large amount of data, rules and constraints behind the data can be automatically discovered and utilized for accurate prediction^23^. However, in scientific applications such as mitosis detection, the amount of data is inherently limited and incorporating known constraints as DOL can be advantageous over purely data driven approaches. In future, we would like to explore the possibilities of DOLs further in various scientific domains.

### Supplementary Information

Supplementary Information.

## Data Availability

The source code of GNN-DOL can be found at https://github.com/95-HaishanZHANG/GNN-DOL. C2C12 dataset is available at https://osf.io/ysaq2/. The additional annotations are available from Prof. An-An Liu (liuanantju@163.com) upon request.
