# Supplementary material for: Differentiable optimization layers enhance GNN-based mitosis detection
Source: Sci Rep. 2023 Aug 31;13:14306. doi: 10.1038/s41598-023-41562-y (PMC10471751; doi:10.1038/s41598-023-41562-y)
Supplement: Supplementary file 1 — Supplementary Information. [file 41598_2023_41562_MOESM1_ESM.pdf]

Supplementary Information: Differentiable optimization layers enhance GNN-based mitosis detection

Haishan Zhang, Dai Hai Nguyen and Koji Tsuda

August 8, 2023

Algorithm S1 Mitosis detection using GNN-DOL: Given two images at frame t and frame $t+1$, it predict whether a cell in frame t is the parent cell of cells in frame $t+1$ or not.

Input: Image sequence from frame 1 to frame T, pre-trained model of UNet, the trained GNN-DOL model $G(\theta)$ with trained parameters θ

Output: Binary correspondence matrix where its element equals to 1 if two cells correspond

```
for t = 1, 2, ..., T − 1 do
    Acquire two images at frame t and frame t + 1 with m cells and m′ cells respectively.
    Construct two graphs G_t and G_{t+1} with two kinds of feature matrix V and E where V_t = [(x_i, y_i, v_i)]_{i=1}^m, E_t = [(x_s, y_s, x_t, y_t)], s ∈ [1, m], t ∈ N(s), V_{t+1} = [(x′_i, y′_i, v′_i)]_{i=1}^{m′}, and E_{t+1} = [(x′_s, y′_s, x′_t, y′_t)], s ∈ [1, m′], t ∈ N(s), where N(s) is the the adjacent nodes of s.
    for i = 1, 2, ..., m do
        Update node features and edge features using message passing:
        v̄_i = MLP(v_i, Σ_{j=1}^m w_ij e_ij) where w_ij = Σ_{k∈N(i)} Σ_{l∈N(j)} ||e_ik − e_jl||^2, and j ∈ [1, m′].
        ē_ij = MLP(e_ij, v_i, v_j)
    end for
    Calculate mm′ × mm′ pairwise similarity matrix Q.
    Solve the quadratic programming to get the correspondence matrix Z_t with eq.
end for
return Correspondence matrix sequence [Z_t]_{t=1}^{T−1}
```

Where, in LaTeX notation:

- Construct two graphs G_t and G_{t+1} with two kinds of feature matrix $\mathbf{V}$ and $\mathbf{E}$ where $\mathbf{V_t} = [(x_i, y_i, \mathbf{v}_i)]_{i=1}^{m}$, $\mathbf{E_t} = [(x_s, y_s, x_t, y_t)], s \in [1, m], t \in N(s)$, $\mathbf{V_{t+1}} = [(x'_i, y'_i, \mathbf{v}'_i)]_{i=1}^{m'}$, and $\mathbf{E_{t+1}} = [(x'_s, y'_s, x'_t, y'_t)], s \in [1, m'], t \in N(s)$
- $\bar{\mathbf{v}}_i = \mathrm{MLP}(\mathbf{v}_i, \sum_{j=1}^{m} w_{ij}\mathbf{e}_{ij})$ where $w_{ij} = \sum_{k \in N(i)} \sum_{l \in N(j)} \|\mathbf{e}_{ik} - \mathbf{e}_{jl}\|^2$, and $j \in [1, m']$.
- $\bar{\mathbf{e}}_{ij} = \mathrm{MLP}(\mathbf{e}_{ij}, \mathbf{v}_i, \mathbf{v}_j)$
- Calculate $mm' \times mm'$ pairwise similarity matrix Q; return $[Z_t]_{t=1}^{T-1}$
